# Characterizing Antimicrobial Use in the Livestock Sector in Three South East Asian Countries (Indonesia, Thailand, and Vietnam)

**DOI:** 10.3390/antibiotics8010033

**Published:** 2019-03-25

**Authors:** Lucy Coyne, Riana Arief, Carolyn Benigno, Vo Ngan Giang, Luu Quynh Huong, Saharuetai Jeamsripong, Wantanee Kalpravidh, James McGrane, Pawin Padungtod, Ian Patrick, Luuk Schoonman, Erry Setyawan, Ady Harja Sukarno, Jutanat Srisamran, Pham Thi Ngoc, Jonathan Rushton

**Affiliations:** 1 Epidemiology and Population Health, University of Liverpool, Neston CH64 7TE, UK; ianpatrick4229@gmail.com (I.P.); J.Rushton@liverpool.ac.uk (J.R.); 2 Center for Indonesian Veterinary Analytical Studies, Bogor 16310, Indonesia; rianaarief83@gmail.com; 3 FAO Regional Office for Asia and the Pacific, Bangkok 10200, Thailand; carolynbenigno@gmail.com (C.B.); Wantanee.Kalpravidh@fao.org (W.K.); 4 FAO Country Office for Vietnam, Hanoi, Vietnam; Ngangiang.Vo@fao.org (V.N.G.); Pawin.Padungtod@fao.org (P.P.); 5 National Institute of Veterinary Research, Hanoi, Vietnam; lqhuongvet@yahoo.com (L.Q.H.); minhngoc27169@gmail.com (P.T.N.); 6 Department of Veterinary Public Health, Faculty of Veterinary Science, Chulalongkorn University, Bangkok 10330, Thailand; Saharuetai.J@chula.ac.th (S.J.); jutanatmilk@hotmail.com (J.S.); 7 FAO Country Office for Indonesia, Jakarta 10250, Indonesia; James.McGrane@fao.org (J.M.); luuk.schoonman@gmail.com (L.S.); erry.setyawan@gmail.com (E.S.); adiharja@gmail.com (A.H.S.); 8 Agricultural and Resource Economic Consulting Services, Armidale, NSW 2350, Australia

**Keywords:** antimicrobial, antibiotic, antimicrobial resistance, antimicrobial use, economics, Vietnam, Indonesia, Thailand, framework, policy

## Abstract

A framework was developed to characterize the antimicrobial use/antimicrobial resistance complex in livestock systems in Indonesia, Vietnam, and Thailand. Farm profitability, disease prevention, and mortality rate reduction were identified as drivers toward antimicrobial use in livestock systems. It revealed that antimicrobial use was high in all sectors studied, and that routine preventative use was of particular importance to broiler production systems. Misleading feed labeling was identified as a hurdle to the collection of accurate antimicrobial use data, with farmers being unaware of the antimicrobials contained in some commercial feed. Economic analysis found that the cost of antimicrobials was low relative to other farm inputs, and that farm profitability was precariously balanced. High disease and poor prices were identified as potential drivers toward economic loss. The research indicates that antimicrobial use in small-scale poultry production systems improves feed conversion ratios and overall productivity. However, data were limited to quantify adequately these potential gains and their impacts on the food supply. During the study, all countries embraced and implemented policies on better management of antimicrobial use in livestock and surveillance of antimicrobial resistance. Future policies need to consider farm-level economics and livestock food supply issues when developing further antimicrobial use interventions in the region.

## 1. Introduction

### Antimicrobial Resistance as a One Health Challenge

There has been a continual increase in the human population from 2.5 billion in 1960 to 7.53 billion in 2017 with growth projected to reach 10 billion by 2050. Much of the future population expansion is predicted to be in low income and middle income countries (LMICs) [1,2]. In parallel, there has been increasing urbanization with a greater proportion of the global population now residing in urban areas [3]. This transition has been linked, in part, with rising household incomes and is accompanied with growing demand for animal source proteins [4].

The increasing demand for animal source proteins has been met through a global shift toward more intensive livestock production systems with a focus on the monogastric species (predominantly pigs and poultry). These systems rely heavily on the availability of cheap feed grains and oilseed cake, which are in limited supply in some LMICs. This raises doubts over the economic sustainability of such a food system approach. Highly integrated and intensive production systems require antimicrobials to ensure animal health and maintain productivity and profitability. Thus, alongside this intensification of livestock production, there has been an accompanying increase in antimicrobial consumption and a consequential increase in antimicrobial resistance (AMR) [5].

The global concerns over AMR are well documented with Lord O’Neill estimating a potential human mortality in the region of 10 million by 2050 with an accompanying economic burden of 100 trillion USD [6]. AMR has its origin in the unregulated use of antimicrobials in the human health, veterinary medicine, and production sectors. This use exerts selection pressure on pathogen populations that encourage the development of resistance and exchange of resistance genes. The threat AMR poses to human and animal health has resulted in growing international pressure to minimize the prescribing of antimicrobials and ensure that use is prudent in both human and veterinary medicine [7,8,9,10].

The zoonotic pathways for the spread of resistance from organisms existing within animal populations to those of significance to human health are well recognized and known to result from the use of antimicrobials in livestock. Consequently, antimicrobial use in food-producing animals is considered to present a risk to human health [11]. While this risk is presently unquantifiable in a precise manner, isolated incidents of such transfer are widely described in the literature [8,12,13,14,15]. Research into intensive agricultural systems have identified that the intestinal microbiota of food producing animal species can act a source of resistant bacteria for those working and living in close proximity [16,17,18], while multi-drug resistant bacterial zoonosis are frequently described and may represent a major threat to public health [19,20,21]. In light of these early indicators of risk, it has been considered prudent that restrictions are placed on the use of antimicrobials in both veterinary and human medicine, with the aim of slowing the emergence of resistance [12,22,23].

A call has been made to create an intergovernmental panel for AMR [24] and, more recently, the challenges of AMR have been expressed as a quintessential One Health issue [25]. These calls highlight the need for a multi-government, multi-sectoral, and multi-species approach. To support these aspirations, innovative ways for surveillance and monitoring of antimicrobial use and AMR are required to address major gaps in knowledge on the use of antimicrobials in livestock and aquatic farmed species. This innovation needs to be led by, or at least include, the private sector. A broader analysis is needed in which understanding antimicrobial use includes analyzing the institutional, social, and economic environments within which decisions are made [26].

AMR and the livestock and aquatic species should be viewed as an antimicrobial use/AMR complex due to its multi-dimensional nature with influence on: food production and productivity, pathogen management, AMR change, and environmental and human health impacts. Interpretation of AMR findings requires a more complete understanding of the inputs to the system, antimicrobial use, and antimicrobial residues in the environment and animal products. This challenge starts with basic building blocks with a focus on areas of likely highest risk. Surveillance, research, and interventions must all be designed to be applicable to the context of the specific situation in each country [27].

In South-East Asia, a large number of antimicrobials are used in the livestock sector, and studies suggest that AMR may be widely prevalent [28]. Weak or non-existent regulatory frameworks governing antimicrobial use, sub-optimal enforcement, and compliance with existing guidelines, low levels of AMR awareness, and inadequate commitment to responsible antimicrobial stewardship are driving the development of AMR. Regionally, AMR mitigation measures in the livestock sector lag behind the human health sector, and there remain gaps in data and information across the pharmaceutical and food sectors. These knowledge gaps limit the assessment of antimicrobial use and, therefore, create difficulties in determining levels and flows of residue and AMR across the food system. The current study developed a framework to characterize and describe the antimicrobial use/AMR complex in three key countries in South East Asia including Indonesia, Thailand, and Vietnam. The World Bank classifies Indonesia and Vietnam as lower-middle income countries while Thailand classifies as an upper-middle income country [29]. Some key statistics related to the economy and agricultural sectors in each country are shown in Table 1.

The framework has three phases to explore the antimicrobial use/AMR complex in livestock systems. First, a detailed literature review identified existing knowledge and defined the major questions on the costs and benefits of antimicrobial use. Second, detailed case studies were undertaken to address some of these knowledge gaps described. In the final phase, a detailed analysis and discussion about the case study and literature review results was presented.

The application of the framework has allowed the development of informed policy suggestions and identified information gaps and priority areas for future research, which this paper summarizes. An exploration of the economic importance as of antimicrobials through the case studies has identified the challenges, and potential negative consequences, of policy to reduce use on small commercial livestock systems. As previously outlined, it is essential that AMR is addressed from a One Health perspective and such farmers’ livelihoods need to be considered, in parallel with the human and animal health concerns from antimicrobial use in livestock. Thus, the framework highlights the importance of collecting accurate farm productivity data and undertaking economic assessments in any interventions to reduce antimicrobial use in LMICs.

## 2. Results

### 2.1. An Overview of Livestock Production Systems

#### 2.1.1. Pig Production

Pork is an economical and sustainable protein source [30]. Pigs are typically reared in intensive systems with many features being uniform internationally such as indoor production, slatted systems, the use of commercially mixed feed, and sow crate systems [31]. Pig production is socially and economically important in Vietnam where three-quarters of meat consumed is pork. Pork is less important in Indonesia where, due to religious beliefs, it is only consumed by around 13% of the population [32,33]. An overview of the pig production sectors is presented in Table 2.

Detailed case studies in Vietnam and Thailand addressed the antimicrobial use/AMR complex in the pig sectors. There were many parallels between the production systems on the case study farms in these countries where the housing and management systems are similar but with marked differences in the scale of production. Overall, the farms observed in Thailand were on a much larger scale and were more likely to be part of an integrated supply chain than those in Vietnam. Additionally, pig production was of a greater economic significance to the individual households interviewed in Thailand when compared with Vietnam. An overview of the production systems from the case studies are shown in Table 3.

#### 2.1.2. Broiler Production

Globally, broiler production is undergoing a process of intensification with the increasing dominance of integrated supply chains [36]. Broiler meat is of a high economic importance across all the case study countries with the greatest importance found in Thailand and Indonesia. Indonesia has a large domestic market for broiler meat, which is estimated to be 87% of all meat consumed [37]. Thailand, in addition to national markets, has a large export market, which is of great economic significance within the region [38]. An overview of the broiler production sectors is shown in Table 4.

There is an ongoing transition across Asia from traditional open housing systems to modern closed and automated systems. This transition has been somewhat accelerated due to the outbreaks of Highly Pathogenic Avian Influenza (HPAI) in the early 2000s [36,45]. The type of housing system is where the main contrasts are observed between broiler production in Thailand, Indonesia, and Vietnam. Thailand has undergone a more rapid transition toward intensive production with a modern, integrated, and intensive broiler industry producing birds for export. The majority of production is from closed systems with automated and controlled environments [38]. In contrast, the transition has been slower in Indonesia and Vietnam with the majority of farms still being smaller scale, open housing with low biosecurity, high mortality rates, and disease signs.

#### 2.1.3. Aquaculture Production

Aquaculture production systems are of growing importance, as global demand for fish continues to rise [46]. This increased demand has been met by a move toward more intensive aquaculture systems with this transition being most advanced in the shrimp sector. The Asian continent is of great significance to international shrimp supply and accounts for 85% of global aquaculture production. However, the emergence of Early Mortality Syndrome (EMS) in 2011 has resulted in a fall of 13% in international shrimp production with Vietnam and Thailand suffering heavy losses [47]. An overview of the aquaculture production sector is shown in Table 5.

### 2.2. Antimicrobial Use Policy and Surveillance

As in many other LMICs, there is currently no requirement for a prescription for the use of antimicrobials in animals or humans in any of the case study countries [52]. However, Thailand has led the way in applying stricter regulations on antimicrobial use in livestock with measures including a ban since 2015 on the use of antimicrobials for growth promotion. In 2017, the antimicrobial classes identified by the World Health Organization (WHO) were identified as being the highest priority, critically important antimicrobials (HP-CIAs) for human medicine, which are only available in veterinary prescription (polymixins, third and fourth generation cephalosporins and fluoroquinolones) [53,54,55]. In comparison, Vietnam and Indonesia currently have fewer restrictions on the use of antimicrobials in animals, but both countries prohibited the use of antimicrobials for growth promotion at the beginning of 2018. If the development of antimicrobial use policy is considered to be a stepwise process, with a prescription-only status for antimicrobials for use in animals being the final goal. Then Indonesia and Vietnam started the process two years after Thailand. For example, the introduction of a national strategy for AMR was launched in Thailand in 2015 while the equivalent strategies were not initiated in Indonesia and Vietnam until 2017 [54,56,57].

Stricter legislation on antimicrobial use may be seen as a positive step in the global fight against AMR, which, to be truly effective, requires the development of enforcement capacity. For example, despite a more extensive infrastructure and tighter control over antimicrobial use in Thailand, researchers have described black market and illegal use of antimicrobials in livestock [58,59], while antimicrobial residues have been identified in eggs and meat from retail outlets in Vietnam despite regulation of maximum residue limits [60,61]. In addition, the case studies identified that farmers were reluctant to adopt new legislation. The majority (>75%) of farmers believed that there was an economic advantage to antimicrobial use in livestock and reported that antimicrobials contributed toward greater farm profitability and lower mortality rates in livestock. In addition, 27% of Indonesian broiler producers in the case study stated that an improvement in the growth rates of birds motivated their use of antimicrobials. Data around the growth and production response to antimicrobials are poorly documented in the types of systems studied and the assumption that there will be no impact on productivity with a change in use needs more careful research.

In Indonesia and Vietnam, antimicrobial use surveillance in animals are either absent or restricted to small surveys or national estimates such as the data presented in the OIE report on global antimicrobial use [62]. The accuracy of national estimates on antimicrobial consumption is often questionable due to the large number of assumptions made to collate these data. In comparison, Thailand has some data available on national antimicrobial consumption in livestock. The Animal Health and Products Association (AHPA), which is the trade association for veterinary pharmaceuticals, has collated antimicrobial sales data to estimate antimicrobial use. Since 2013, the AHPA has used the European Surveillance for Veterinary antimicrobial Consumption (ESVAC) metric of mg of antimicrobial per population correction unit (mg/PCU). These data identified pigs to be the species with the highest consumption of antimicrobials with a value of 238 mg/PCU in fattening pigs compared to 16 mg/PCU in broiler chickens [63].

Indonesia has an established animal health surveillance system as the ‘integrated animal health information system,’ which is referred to as iSIKHNAS. This is a farmer-based reporting system for disease, animal population, and animal treatments [64]. The system also requires that all government veterinarians and para-veterinarians report any medicine usage [65]. This program has proved to be an effective method for capturing antimicrobial use data for small beef production. However, it rarely captures data on commercial broiler or pig farms [64,65].

### 2.3. Antimicrobial Use Behaviors

#### 2.3.1. Pig Production

The focus of the case studies in Thailand and Vietnam was the small-medium commercial pig sector. The literature review highlighted this sector as having relatively high antimicrobial use in comparison to backyard or large integrated production systems [20,32,66]. In addition, this sector may present a larger human population employed along the supply chain, and, thus, is at risk of exposure to antimicrobials with more labor-intensive systems and manual slaughterhouse facilities. These farms are also more likely to supply local wet markets, which may present a greater food safety risk when compared with the more formal retail supply chain [67].

**Figure 1:**
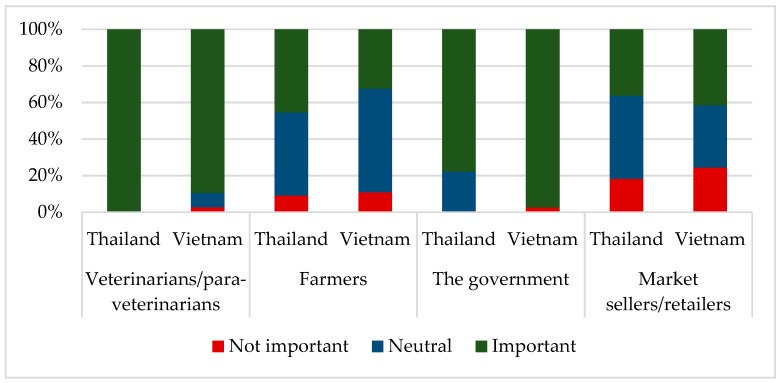
Farmer attitudes to the roles of different actors in monitoring the prudent use of antimicrobials in pigs.

In Asian LMICs, antimicrobials are freely available over the counter for use in both humans and animals [58,68]. Therefore, many farmers administer antimicrobials without the guidance of a veterinarian and source products directly from pharmaceutical companies, drug sellers, or pharmacies. However, the survey found that the majority of the respondents in Thailand and Vietnam identified the veterinarians to be the most important people in monitoring the prudent use of antimicrobials in pigs. In parallel, there was a shared opinion that the government was important in monitoring the responsible use of antimicrobials in pigs. Conversely, many farmers did not consider themselves accountable for monitoring the prudent use of antimicrobials in pigs. Farmer attitudes for monitoring the prudent use of antimicrobial nature in pigs are shown in Figure 1.

**Figure 2:**
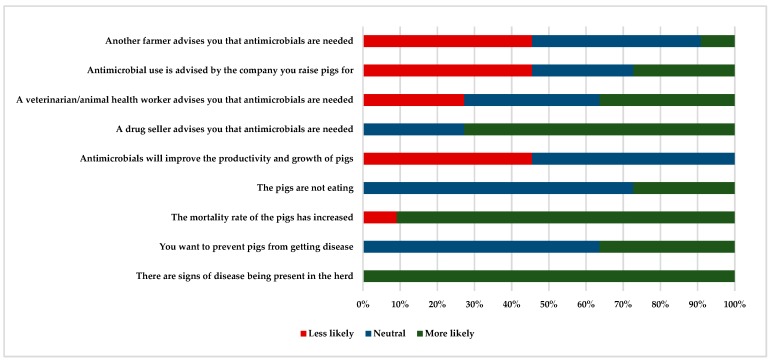
The behavioural influences behind antimicrobial use in a case study on antimicrobial use on pig farms in Thailand (*n* = 11).

All of the pig producers in the case studies in Thailand and Vietnam identified that there was an economic advantage to the use of antimicrobials in pigs. The most frequently volunteered justifications were that antimicrobials improved farm profitability, reduced mortality, and improved pig productivity (Table 6). Influences of antimicrobial use in Thailand included the presence of disease, increasing mortality rates, and drug seller advice. No farmers reported that antimicrobials were used to improve farm productivity, but they reported that antimicrobial use for growth promotion was a justified practice (82%, *n* = 11 in Figure 2).

**Figure 3:**
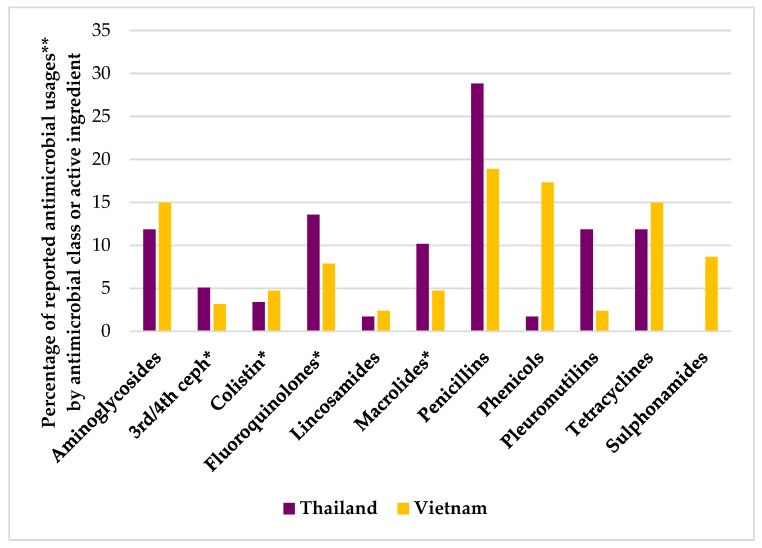
Reported antimicrobial usages by active ingredient (%) in a case study into antimicrobial use in pig production in Thailand and Vietnam. *Antimicrobial class classified by the WHO as the highest priority as critically important antimicrobial (HP-CIA). **A ‘reported antimicrobial usage’ was considered the use of an antimicrobial active ingredient or the use of the same active ingredient in a different formulation, as reported by the farmer. In the case of a combination of antimicrobial product, each active ingredient was counted as differently reported antimicrobial usage.

The case studies also sought to collect quantitative data on antimicrobial use by indication, formulation, and active ingredient (Table 7). The penicillin, tetracycline, and aminoglycoside classes were the most commonly reported antimicrobial usages across pig production systems in both countries. However, there were some contrasts in antimicrobial use behaviors observed. For example, the phenicol class accounted for around a third of reported antimicrobial usages in Vietnam when compared with minor use (1.7%) in Thailand. Antimicrobial use of the HP-CIA classes was more frequent in Thailand (32%) when compared to Vietnam (21%) (Figure 3).

The collection of these antimicrobial use data presented some challenges. First, some of the commercially mixed feed on farms did not include information on the ingredients or antimicrobial content on the feed labels. Therefore, in-feed antimicrobial use may be under reported. Second, the bin collection method used in the Vietnamese case study proved to be an inefficient method of collecting quantitative antimicrobial use data. There was poor understanding of the methodology by many participants and, consequently, few farms retained antimicrobial packaging for the full six-week study period.

#### 2.3.2. Broiler Production

The focus of the case study in Indonesia was the small-medium commercial broiler sector. The literature review identified that the Indonesian broiler sector relies heavily on antimicrobials for routine disease prevention programs [65]. In addition, while the industry has undergone rapid intensification, the majority of Indonesian farms are still open housing systems. These systems pose a significant disease risk and are considered to have low levels of farm biosecurity [39], but are probably more aligned to the economic reality of producing chicken in Indonesia.

**Figure 4:**
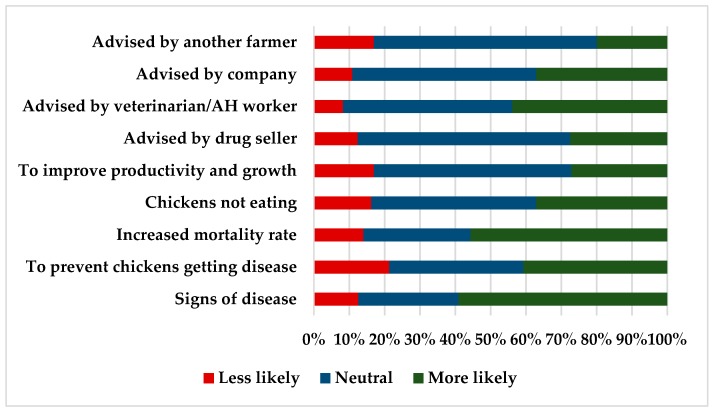
The behavioral influences behind antimicrobial use in a case study on antimicrobial use on broiler farms in Indonesia (*n* = 419).

The influences behind antimicrobial use behaviors in the small-medium broiler sector in Indonesia identified that the active presence of disease, increasing mortality rates, and an aspiration to prevent disease were major drivers of antimicrobial use on the case study farms. In addition, in spite of a ban on the use of antimicrobials for growth promotion, 27% (*n* = 419) of respondents identified that an improvement in productivity and growth was a motivation toward antimicrobial use. The majority of farmers (44%) considered that advice from veterinarians or para-veterinarians would drive antimicrobial use and 88% identified that veterinarians played an important role in monitoring the prudent use of antimicrobials. Equally, the dominant opinion was that the government (85%) and farmers (80%) played an important role in monitoring antimicrobial use in broilers in Indonesia. The behavioral influences behind antimicrobial use are shown in Figure 4.

Overall, 81% of farmers identified that there was an economic advantage in using antimicrobials in broilers with increased productivity and healthier chickens being the most commonly cited reasons (Table 8). In parallel, the majority of farmers (88%) felt that disease had a negative effect on farm profit margins. However, 21% did not record any production data. When production data were collected, mortality rates (23%), body weight (22%), and sale price (12%) were most frequently recorded.

The case study sought to describe baseline knowledge of farmers on the issue of AMR. In an open response question, only 45% (*n* = 419) of broiler farmers were able to correctly define AMR. The most frequent definition was that AMR is *‘resistance to drugs.’* However, some offered a more general description that AMR *‘caused treatment to be ineffective’*. However, 56% of farmers identified that AMR was a major challenge in human medicine and an equal number felt that antimicrobial use in broilers might affect the health of consumers.

#### 2.3.3. Aquaculture Systems

Studies show that antimicrobial use is widespread in aquaculture systems in Indonesia, Thailand, and Vietnam [65,69,70,71]. Tetracyclines, fluoroquinolones, sulphonamides, and penicillins are important antimicrobial classes used in the sector [65,69,71] and are most frequently sourced over the counter from feed supply shops. Since there are limited medicated feeds available, farmers manually mix antimicrobials into feed with a potential risk of inaccurate dosing and concerns over the public health consequences from exposure to active ingredients [65,69].

There are significant concerns over the presence of antimicrobial residues in fish and the potential for the transmission of antimicrobial resistant bacteria to humans through both food and the environment [72,73,74]. Multi-drug resistance has been identified in *Escherichia Coli* and *Vibrio* from environmental samples and shrimp for sale through retail outlets in Thailand [73,75], while *Pangasius* catfish production is considered to represent a significant ecological risk from antimicrobial residues and resistant bacteria in the environment [76,77]. Integrated livestock-fish production systems, whereby manure is used as a feed for fish, are an economically important sector through Asia. However, these systems are under scrutiny for the potential risks to human health from antimicrobial residues [78]. For example, antimicrobial contamination of the environment and fish has been found to occur as a consequence of chlortetracycline administration to chickens in integrated chicken-fish production systems [79].

The discovery of antimicrobial residues in exported farmed shrimp has resulted in Japan imposing importation restrictions on Indonesia and Thailand [74]. Consequently, Thailand has introduced policy banning the use of medicated feeds in aquaculture, while Indonesia only permits the tetracycline, macrolide, and fluoroquinolone classes to be used in aquaculture systems [52,65]. Additionally, Vietnam has policy to restrict the use of enrofloxcin in aquaculture. However, a study by Thi Kim Chi et al. (2017) identified the sale of enrofloxacin in feed shops in two different provinces in Vietnam, which cast doubt over the enforcement of such legislation. Thus, while further exploration of aquaculture systems was beyond the scope of this framework, the sector presents a number of concerns with regards to antimicrobial resistance, residues, and policy enforcement. This is an area that warrants further research.

### 2.4. The Economic Drivers for Antimicrobial Use

An important step in defining the role of antimicrobials in pig and broiler production systems is to understand the costs and benefits to individual farmers. Ultimately, the role of the economic analysis is to be able to compare production under different input scenarios and identify the misallocation of resources through a search for optimal resource use under a set of prices for inputs and outputs. This search for optimal use has to compare production with and without antimicrobials and to compare the effect of using alternative non-antimicrobial interventions on production. The following presents an economic analysis of the Vietnamese and Thai pig production systems as well as the Indonesian broiler sector, but the scope of the study did not allow economic assessments of alternative levels of antimicrobials use and alternative interventions to antimicrobials in these systems. This requires longitudinal work as described by Carrique Mas and Rushton (2017) in order to refine the generic recommendation of ‘reduction in antimicrobial use’ toward a set of guidelines on how to reduce antimicrobial use with minimal impact on livestock production, profitability, and livelihoods and maximum impact on AMR management and general pathogen control [80].

The ability to undertake more detailed economic analyses was limited by the lack of detailed on-farm production and cost data. In these small-to-medium scale enterprises, farmers do not routinely collect any such data, which reflects the relative costs of data collection and the benefits gained from such activities in small production units. The project was able to provide initial estimates of the importance of antimicrobial costs to the economic viability of these small-to-medium scale production systems.

#### 2.4.1. Vietnam

The economic analysis in Vietnam used the data from those farms who had sows in 36 rather than the 40 farms in the entire survey. The management and economic landscape of farms that specialized in fattening (three of the 40) was very different to those who managed a farrowing to fattening production system. In addition, a recent decline in price led to some of these fattening farms not purchasing weaners for fattening in the previous 12 months. One farm produced 900 fatteners and was regarded as an outlier in this study.

The gross margin analysis, difference between output and variable costs, indicates that returns have been poor during the time of this survey with two out of the three scenarios indicating losses to the farmer. The result supports the perceptions of the study team and enumerators that the pig sector was facing a difficult economic environment during this time.

Medicines and antimicrobials were only 2% of costs in the pig production system, which is an important result when considering policies on pricing as a way to manage antimicrobial use. As expected, feed was the most significant cost and, therefore, the input of greatest concern by farmers. The low cost of antimicrobials could well mean that farmers are not in a conscious process of searching for optimal antimicrobial use around a classic production function of output. Rather the use, as shown in the behavioral aspects, is in response to a mix of managing impacts of disease and health issues that support production. It is, likely, best described as a risk management strategy in an uncertain environment. While the internal farm costs are relatively low with current pricing, the potential externalities of poor on farm antimicrobial use leading to AMR risks to humans is not properly accounted for in the price. Further work is required to estimate the product development costs, the effects of AMR on future pig production and disease control, or the present and future effects on human health.

#### 2.4.2. Thailand

With regard to the survey of pig farmers in Thailand, nine out of the 11 farmers surveyed had large herds. The data showed that they were selling an average of 334 pigs, probably fatteners, which would provide a weekly gross income of $60,000 USD. Investing in medicines and, more specifically, antimicrobials turned out to be a very minor cost. Investing $2.03 USD to maintain an asset of $180 USD, most farmers regarded the value of a fattener as good value for money even if the actual benefits are unclear. Likewise, a preventive medication of $2.07 to have potential health benefits to their breeding sows may seem to be a good investment.

Farmers were also requested to list the antimicrobials used, the active ingredients, cost per pack, and which pig age group they were used on. Once again, these data were very difficult to recall and some farmers could not, or would not, provide estimates. While routine antimicrobials could be estimated, it was not possible to estimate what antimicrobials were actually given as disease treatments. The antimicrobials were listed but farmers found it difficult to allocate costs to pigs in a cycle or year, so another measure was developed to show the relative importance of antimicrobials to the farming system. The conclusion is that, once again, antimicrobials are a very small cost to the farmer. For every kilogram of pig on the farm at the time of the survey, the farmers on average expect to spend, or at least identified costs of, $0.06/kg live weight on antimicrobials.

#### 2.4.3. Indonesia

Gross margin data and antimicrobial costs per cycle were not able to be estimated from the survey data. The economic analysis, therefore, considered some alternative measures. First, a more simplified analysis that assesses whether or not there are differences in medicine costs between farmers’ performance index (Performance Index = ((weight at sale [gms]/age at sale [days]) × liveability [%])/FCR × 10) results in their last broiler cycle. The study identified the possibility that the higher the performance index is, the lower the use of medicines are. The farmers with the highest performance index (highest 25% of farmers) invest, on average, Indonesian Rupiah350/bird (US$0.02/bird) on medicines. The range for the other farmers is between IDR390 and IDR405/bird. While this may be a small difference in costs per bird when extrapolated over the whole flock, it may be significant.

The survey collected information concerning the number of days that antimicrobials were used on the most recent batch of broilers. This may be used as a proxy for antimicrobial usage on the farm. It may be that, the more days that antimicrobials are used, the more antimicrobials are actually used. Although this does not provide insights into the reasons for use or even the types of antimicrobials used, it still may provide useful information concerning the extent of use. The analysis showed that, with regard to farm size, there is a slight difference between smaller farms and the larger farms with respect to the number of days that antimicrobials are used. The larger farms may tend to use less antimicrobials than the smaller farms.

Another useful measure is the correlation between the average performance index and the number of days that antimicrobials are used. There may be a positive relationship between antimicrobials and the index. The more days that antimicrobials are used, the higher the performance index is. The context of these results is that antimicrobial use in this system tends to be a preventative measure with advice from the contract company to use antimicrobials if bad (hot or cold) weather is expected and fear of respiratory disease. In addition, farmers, particularly independent producers, often give antimicrobials on the day the one-day old chick arrives since this is a critical point in the production process. There are often doubts about the strength of the newly arrived bird to survive the transition from the hatchery to the farm.

## 3. Discussion

### 3.1. Drivers for Antimicrobial Use in Livestock

The small commercial livestock sector runs on tight profit margins and producers are highly susceptible to changes in market prices. This economic vulnerability has been shown through the Vietnamese pig industry since the sector has experienced a turbulent economic scene since early 2017 with falling pig prices from an oversupply of pork and a cessation of exports to China. As a consequence, it has been estimated that around 30% of smaller pig farms have gone out as a business in Vietnam [81,82]. With farms balanced precariously between profitability and loss, it is essential that the economic landscape of antimicrobial use is mapped to ensure a future sustainable food supply.

Keeping detailed farm records on livestock production can enable farmers to identify which areas of their business may benefit from additional investment and where losses may be occurring. These data are also required to present to financial lenders for proof of reinvestment potentials on the farm [83]. However, either many of the farmers in the case study did not keep records or production data records were limited. There is scope for further engagement of farmers to review how data are collected and how benefits can be generated in terms of improved management and biosecurity to improve farm profitability, manage risk, and reduce variation.

Farm profitability, disease prevention, and reducing mortality rates were identified as drivers towards antimicrobial use in livestock systems. In broiler systems in Indonesia, the use of antimicrobials in feed were identified as a method of compensating for the significant disease burden seen in open housing systems [84]. While the Indonesian broiler sector is undergoing a process of intensification, with a move towards closed automated housing systems, this shift is predominantly in the large integrated sector [39]. Therefore, at present, the small-scale broiler sector presents a number of challenges concerning disease prevention and there is concern that any regulation to restrict access to antimicrobials may result in some farms being unable to economically produce broilers.

The use of medicated feed to prevent disease is a frequent practice in global livestock systems [85]. This behavior was identified in all of the case study countries, but it was potentially under-reported in the pig sector. For example, through the Vietnamese case study, some of the commercial mixed feed was found to be missing feed ingredient listings while evidence from the literature suggests that over half of commercial pig feed contains antimicrobials [86]. Issues with misleading and imprecise labelling of livestock feed has been identified in other studies in the region and improving the regulation of feed labelling is a priority area for AMR policy in South East Asia [52,86,87].

The use of antimicrobials for disease prevention is a contentious issue and there is increasing pressure to discontinue this practice internationally [5,8,88]. There has been a move in many high income countries (HICs) for chicken broiler production that do not use antimicrobials. This has has been driven, in part, by food companies seeking a market for livestock products from antimicrobial free systems, but there are welfare implications for these systems. For example, chicken broiler systems that do not allow drug use for the control of coccidiosis in addition to prohibiting antibiotic use can suffer disease consequences [89]. It was reported that broiler production in Thailand by one of the major integrator companies was producing broiler meat for export under antimicrobial-free conditions [90].

The easy availability of antimicrobials over the counter and from a range of sources results in antimicrobial costs remaining consistently low throughout Asia [68]. The study identified that, in pig and poultry systems, the cost of antimicrobials was low relative to other inputs. The antimicrobial use was driven by response to advice from others and by the presence and risk of disease or an animal health issue. A policy to increase antimicrobial prices may generate a rapid decline in use. However, there are concerns that increasing costs may incentivize farmers to source antimicrobials from the black market. In Thailand, an increasing price of feed containing premixed antimicrobials has resulted in some farmers purchasing antimicrobial active ingredients to mix into feed manually in spite of regulations prohibiting such behaviors. This practice may pose a risk to human health from farmer exposure to active ingredients in the mixing process [58]. Therefore, it is essential that the potential for such behaviors be considered in any future regulation of in-feed antimicrobial use to mitigate these risks to farmers. The previously mentioned example shows that regulation alone cannot be considered sufficient to control antimicrobial use behaviors. There also needs to be sufficient resources to enforce policy.

A ban on antimicrobial growth promoters in Denmark was shown to increase the need for antimicrobials for therapeutic indications in the years following the ban [91]. This was believed to be linked with increasing levels of disease previously masked by the use of growth promoters and the associated reductions in productivity. Antimicrobial growth promoters are considered to be particularly beneficial for disguising subclinical disease on unhygienic and older livestock housing systems [92]. Therefore, it seems likely that the antimicrobial growth promoter ban will have an effect on the overall productivity of the livestock sectors, which are concerns echoed by participants in the case studies. It is essential that producers receive sufficient support to seek alternative management routes for preventing disease.

There is a grey area around prophylactic and growth promoting antimicrobial use whereby studies have shown that producers may use antimicrobials for growth promotional effects, but justify this practice as being for disease prophylaxis [93,94]. Dang et al. (2013) identified that high and inappropriate doses of antimicrobials were frequently used in pigs in the Red River Delta region and that course lengths were frequently sporadic. Such irresponsible behaviors may suggest that inappropriate use for disease prevention or potentially growth promotion may still be an issue globally. The case study results support this theory by showing poor farmer awareness of the ban on antimicrobial growth promoters and many considered livestock growth to be a driver for antimicrobial use. Thus, there is a need for further knowledge exchange with producers, veterinarians, and key livestock stakeholders concerning the legislation prohibiting the use of antimicrobial growth promoters. This is likely to pose a significant challenge for the enforcement of national bans and is an area of concern throughout South East Asia, which requires enhanced financial support and resources to properly enforce the legislation [52].

The case study results identified the frequent use of the HP-CIA classes in all livestock groups. There has been increasing international pressure to reduce or discontinue the use of HP-CIA classes in livestock [6,8]. Efforts to address this has varied from voluntary or legislative bans in some European countries [95,96,97] to a requirement for a veterinary prescription for their use in Thailand [54]. The implementation of national action plans on AMR aim to advocate optimal antimicrobial use practices. However, this framework has highlighted that reductions in antimicrobial use are likely to result in significant economic losses and high mortality rates. Therefore, it would be worthwhile to prioritize policies based on risks to human and animal health. One potential route would be to focus initial restrictions on antimicrobial classes considered to be of greater importance to human medicine.

Restricting access to antimicrobials by removing over-the-counter sales has been identified as a potential route to better antimicrobial use in animals in such countries [7,8]. However, there are ongoing concerns that restricting access to prescription-only may result in some communities becoming unable to access antimicrobials [98]. Conversely, evidence from human medicine suggests that, when such policy is implemented, it can have a significant effect on the use of antimicrobials [99]. For example, a study in Chile showed that regulations banning private retail outlets from supplying antimicrobials without a prescription resulted in a significant reduction in national antimicrobial sales [100].

Discussions with pharmacists and drug sellers in Asia have highlighted indiscriminate antimicrobial sales and raised concerns over poor knowledge on AMR and responsible antimicrobial use [101,102,103]. Equally, many veterinarians and physicians who are employed directly along the antimicrobial distribution chain are likely to be rewarded with financial incentives for selling particular antimicrobial products [35,101]. Consequently, Sommanustweechai et al. (2018) surmised that inappropriate antimicrobial use by farmers might be linked with this ease of access and inadequate advice for farmers. Similarly, the farmers in the case studies reported a preference for sourcing antimicrobials directly from pharmaceutical companies and retail drug stores. Thus, identifying pharmaceutical companies and pharmacists/drug sellers as a priority audience for education on responsible antimicrobial use practices. The pharmaceutical industry, feed industry, and pharmacy associations need to be engaged in ongoing work to address antimicrobial resistance and AMR in the region.

At present, there is no easy route through which the sale of antimicrobials through retail outlets and pharmacies can be monitored [58]. This present lack of regulation is likely to be corrected with recommendations in-line with the WHO’s 20th Model List of Essential Medicines, which would categorize certain classes of medicines including antimicrobials as prescription-only [104]. However, even if such legislation is introduced, there is still a continued need to engage the pharmaceutical supply sector in the message on targeted and responsible antimicrobial use.

Poor awareness of AMR concerns by veterinarians have been identified as a challenge for reducing and optimizing antimicrobial use in LMICs [58,85]. Thus, it is essential that the training and advice of veterinarians and para-veterinarians with regards to responsible antimicrobial use is both harmonized and in-line with international prescribing principles for responsible use such as those promoted by the OIE or WHO [8,105].

### 3.2. The Development of the Framework on the Antimicrobial Use/AMR Complex in Livestock

The case studies present data from a small number of small-scale commercial pig and broiler farms in each country. A convenience sampling method was implemented by in-country collaborators and, as such, there is a potential for selection bias [106]. Thus, the results cannot be considered to be representative of national livestock production systems. However, the case study results offer interesting insights into the costs and benefits of antimicrobial use for small commercial producers. There may also be a risk of social desirability bias in the case study results where respondents may report antimicrobial use behaviors that they perceive to be responsible rather than actual practices [107,108]. Nonetheless, the results suggest that any such bias is minimal through the detailed responses provided and that farmers’ reported opinions do not conform with international guidance on responsible antimicrobial use [7,56]. The questionnaire used open questions to try and minimize any social desirability bias by allowing free thought and requiring respondents to propose novel ideas or perceptions not motivated by closed question options [109]. As with the collection of any retrospective data, there is a potential for recall bias. In this context, there was a risk of farmers either under or over reporting antimicrobial use [106]. The training of enumerators for data collection was aimed to minimize this bias by supporting farmers in the recall process such as by using antimicrobial packaging in order to act as a physical reminder.

The bin collection methodology implemented in the Vietnamese case study presented a number of limitations. The farmer was requested to retain all antimicrobial packaging over a six-week period. However, these data were missing for the majority of farms and the farms that did collect packaging frequently collected packaging from non-antimicrobial packaging and did not retain feed labels. Redding et al. (2014) found parallel issues with a lack of understanding by farmers on what constituted an antimicrobial with farmers retaining non-antimicrobial products and disposing of antimicrobial packaging in dairy farms in South America [106]. In addition, the finding that some commercial pig feed labels did not include ingredient listings may have contributed toward inaccurate data collection.

There were issues with collecting a complete set of data from some of the case study farms particularly concerning productivity and economic parameters. Gaps in farm data on productivity and economic costs are a familiar theme across livestock production systems in South East Asia, which limits the scope for conducting detailed analyses into productivity, the financial implications of disease, and the economics of antimicrobial use. Consequently, many of the economic and productivity results reported in the case study are not complete. This issue identifies a hurdle for assessing the effects of any interventions or management changes introduced on farms to reduce antimicrobial use, since, presently, most farms do not collect sufficient productivity data to assess whether such interventions are economically viable.

## 4. Materials and Methods

### 4.1. Framing the Antimicrobial Use/AMR Complex in Livestock in South East Asia

A review of the literature and in-country discussions identified priority livestock sectors, understanding of antimicrobial use and AMR within these sectors, private and public research programs, and the extent of policy support. Pigs and poultry were identified to be the livestock sectors of most economic and social importance across the countries [110]. These sectors provide opportunities to meet the increasing food demand through intensive, integrated, and low-cost production systems. Within these intensive systems, there has been a move toward greater antimicrobial consumption, which has been accompanied with growing concerns for the potential effects on AMR levels in humans, animals, and the environment [5]. Consequently, the following livestock sectors were selected for more detailed investigation into the antimicrobial use/AMR complex.

1. The small-medium commercial broiler sector in Indonesia,
2. The small-medium commercial pig sector in Vietnam,
3. The small-medium commercial pig sector and the larger integrated broiler sector in Thailand.

### 4.2. Case Study Methodology

Quantitative data relating to the economics of antimicrobials, farm productivity, and farm-level antimicrobial use were collected alongside qualitative information on farmer attitudes to AMR, responsibility, and drivers for antimicrobial use through a face-to-face questionnaire in each country. The questionnaire was designed based on a previous questionnaire study on the drivers into antimicrobial use in the UK pig industry and on information gained from previous Knowledge Attitudes and Practices (KAP) surveys undertaken in the South East Asian region by the FAO [111]. In addition, in Vietnam, the bin collection method was also adopted in which farmers were asked to retain antimicrobial packaging over a six-week period in order to quantify antimicrobial use for a defined period of time [106]. Data collection was undertaken using collaborators within the country. An overview of the case study methodologies used in each country is shown in Table 9.

### 4.3. Ethical Approval

Overall ethical approval was granted by the University of Liverpool Veterinary Science Research Ethics Committee, which also required proof of local (country-level) ethical acceptability (Reference number VREC640). In Thailand, the ethical review committee of the department of Health Sciencesfrom Chulalongkorn University granted ethical approval. Since the study did not involve the collection of samples from animals or humans, the research collaborators and local government in Indonesia and Vietnam did not require a specific ethical review. Therefore, documentation mitigating the need for a detailed ethical review was provided from the government livestock departments in each country (Directorate General of Livestock and Animal Health Services (DGLAHS) in Indonesia and National Institute for Veterinary Research (NIVR) in Vietnam). 

## 5. Conclusions and Implications for Policy Development

An overview of the key findings from the framework development to characterize the antimicrobial use/AMR complex in Indonesia, Thailand, and Vietnam and the implications of these conclusions for policy development are presented in Table 10.

A national scheme to promote responsible antimicrobial use in livestock may be a route through which veterinarians and industry stakeholders could be united. This could have an end goal of producing antimicrobial prescribing guidelines for key livestock sectors and advice on alternative methods to prevent disease. Similarly, an independent initiative in the UK has been led by the Responsible Use of Antimicrobials in Animals Alliance (RUMA), which have produced guidelines to support producers in ensuring that antimicrobial use is prudent on livestock farms [112]. Further engagement of veterinarians, independent farmers, livestock production companies, and other key stakeholders is essential if any scheme to promote prudent antimicrobial use is to be successful.

Country-level actions on antimicrobial use should be undertaken with close communications with neighbouring countries as well as at the wider international level. AMR does not respect country borders, which is of particular importance in the South East Asian region where many countries share common borders and have permanent land crossings for importation and exportation. For example, multidrug-resistant *Salmonella species* have been found to be highly prevalent in pigs, chickens, and their products in the Thailand and the Cambodian border provinces [113].

Country-level actions on antimicrobial use should be undertaken with close communications with neighbouring countries as well as at the wider international level. AMR does not respect country borders, which is of particular importance in the South East Asian region where many countries share common borders and have permanent land crossings for importation and exportation. For example, multidrug-resistant *Salmonella species* have been found to be highly prevalent in pigs, chickens, and their products in the Thailand and the Cambodian border provinces [113].

In the case study countries, engaging the private sector in policy development and the implementation of the AMR action plan presents a number of challenges. First, there is strong competition between companies in livestock production. Second, there are great concerns by the private sector over the potential economic effects of reducing antimicrobial use and, third, there seems to be little incentive to reduce antimicrobial use in the present climate [114,115]. However, there is increasing international pressure on private as well as public sectors to address AMR and reduce antimicrobial use in livestock. This is also a strategic aim of AMR national action plans. Thailand is an excellent example of where private sector collaborations can be an effective tool for supporting government actions for AMR. This is shown through the work of the AHPA in publishing estimates of antimicrobial consumption in animals. Similar methodology could be applied to other LMICs in South East Asia. The data collected for the OIE report on antimicrobial use in animals is an excellent starting point for exploring antimicrobial sales by sector [62].

While collecting national farm-level antimicrobial data is likely to be a future aspiration, there is still hope to encourage production companies and farmers, through producer organizations, to improve farm recording of key productivity parameters.

## Figures and Tables

**Table 1:** Key statistics on the economic status and agricultural sectors in Indonesia, Thailand, and Vietnam in 2017.

	Indonesia	Thailand	Vietnam
Population (million)	264	69	93.6
GDP in (billions US$)	932	455	220.4
GDP per capita (US$)	3530	6594	2355
% of the population residing in urban areas	55%	50%	34%
% of population employed in the agricultural sector	31%	49%	35%
Average meat consumption per capita	2.3 kg (pork)	10.4 kg (pork)	30.4 kg (pork)
	7 kg (poultry)	14.5 kg (poultry)	13 kg (poultry)
	1.8 kg (beef and veal)	1.7 kg (beef and veal)	9.9 kg (beef and veal)
	0.4 kg (sheep)	0 kg (sheep)	0.1 kg (sheep)
Average fish consumption per capita	47.1 kg	33.7 kg *	27 kg

* 2016 data. Sources: https://data.worldbank.org/indicator.

**Table 2:** An outline of the key characteristics of pig production in Indonesia, Thailand, and Vietnam.

	Indonesia	Thailand	Vietnam
National pig herd	8 million	9.5 million	27 million
Number of pigs per person	0.03	0.14	0.29
Average farm size	Small herd sizes—around 80% of the national herd are housed on farms with <20 sows	Larger herd size—small production is identified as <500 breeding sows	Much smaller herd size—majority of sow herds have <100 sows
Structure of industry	Predominantly smallholder production	Predominantly large integrated production	Small and medium commercial production More intensive and larger scale production in the south when compared with the north
Pig housing characteristics	Mainly indoor open housing systems	Mainly intensive indoor closed housing systems	Mainly indoor but open housing systems
Market	Domestic consumption except for export to Singapore from one integrated producer	Predominantly domestic consumption and some export of live pigs and chilled/frozen pork to neighboring Asian countries	Pig meat is for domestic consumption
Economic importance and stability	Small domestic market due to the large Islamic population (only 13% of population consume pork)	Thailand has experienced fluctuating pig prices due to an oversupply in 2017 and 2018, DLD stabilized prices at a higher rate in summer 2018	Vietnam has experienced falling pig prices since early 2017, which has resulted in significant contraction in the industry (30% of smaller farms have gone out of business)
References	[33]	[34]	[32,35]

**Table 3:** Demographic information of the sample of pig farms for the case studies in Thailand and Vietnam.

Number of Farms in Sample		Thailand	Vietnam
		11	40
Median number of pigs	Sows and boars	635	5.5
	Piglets (pre-weaning)	1550	20
	Feeding pigs (post-weaning)	3100	40
Type of production system	Farrow to finish farms	85%	55%
	Breeding only farms	5%	18%
	Fattening only farms	10%	27%
Median percentage of annual income from pigs		50%	75%

**Table 4:** An outline of the key characteristics of broiler production in Indonesia, Thailand, and Vietnam.

	Indonesia	Thailand	Vietnam
National broiler flock	3.5 billion	1.1 billion	323 million
Number of broilers per person	13.26	15.94	3.45
Average farm size	Smaller flock sizes with most being 5000–20,000	Large scale production with an average of 70,000 birds in a flock	Average flock sizes <2000 birds
Structure of industry	Commercial integrated production companies producing broilers on small contract farms	Commercial integrated production on large farms Mixture of company farms and some on contract farms	Smallholder and small commercial systems
Broiler housing characteristics	Mainly open housing systems	Mainly closed and automatically ventilated housing	Mainly open housing systems
Market	Broiler meat is for domestic consumption	Thailand has an important export market for broiler meat	Limited export market from larger integrated production
Economic importance and stability	Economic growth in the industry	Economic growth in broiler production in Thailand after recovery from Highly Pathogenic Avian Influenza (HPAI) outbreak	Some economic instability historically to the effects of HPAI. However, there is now growth in the sector.
References	[39,40,41]	[38,42]	[43,44]

**Table 5:** An outline of the key characteristics of aquaculture production in Indonesia, Thailand, and Vietnam.

	Indonesia	Thailand	Vietnam
Total Production in Metric Tons of Live Weight	547,934 in 2012	376,339 in 2013	806,960 in 2013
Export Volume in Metric Tons of Live Weight	~270,000 in 2012	~330,000 in 2013	600,000 in 2013
Dominant aquaculture sectors	Brackish water—Shrimp and milkfish. Freshwater—Tilapia, catfish, carp, and grouper.	Brackish water—Whiteleg shrimp, green mussel, blood cockle, and oyster. Freshwater—Nile tilapia and catfish.	Brackish water—Whiteleg shrimp and tiger shrimp Freshwater—*Pangasuis* catfish
Structure of the industry	80% of the industry is small-scale extensive and semi-intensive cage, net, and pond systems. In addition, there is an emerging intensive cage and net systems sector.	Improved extensive, semi-intensive, and intensive net and cage systems. Intensive systems are dominant for shrimp production for export.	Improved extensive, semi-intensive, and intensive net and cage systems
Market	38% of aquatic production is produced for export and there is a large and rapidly growing export market.	88% of aquaculture products are for the export market.	Shrimp farming accounted for 94% of Vietnam’s export market is 2014. *Pangasuis* catfish contribute toward both the domestic and export market.
Economic importance and stability	Indonesia has made large-scale investments in intensive white leg shrimp farming and exports over half of shrimp produced. The export value > $1 billion.	EMS had a devastating effect on the shrimp sector in Thailand from 2011 through 2014 with high mortality rates. Economic recovery is ongoing.	Aquaculture is very important to Vietnam’s economy and contributed 10% toward the country’s GDP. However, Vietnam has suffered significant losses due to EMS.
References	[47,48]	[47,49,50]	[47,51]

**Table 6:** Farmer volunteered responses to the economic advantages of the use of antimicrobials in pig farms in a case study regarding pig production in Thailand and Vietnam.

	Thailand		Vietnam	
Volunteered responses	No.	Percentage	No.	Percentage
Improves farm profitability	7	41.2%	24	32.0%
Reduces mortality	5	29.4%	21	28.0%
Increases pig herd productivity	2	11.8%	15	20.0%
Antimicrobials are not expensive	1	5.9%	12	16.0%
Reduces culling rates	1	5.9%	0	0.0%
Reduces morbidity	1	5.9%	3	4.0%

**Table 7:** Characteristics of antimicrobials used in a case study in antimicrobial use in pig production in Thailand and Vietnam.

% of Antimicrobial Products Reported to Be Used Routinely on Farms		Thailand	Vietnam
Route of administration	Parenteral	56%	80%
	In-feed	40%	19%
Indication for use	Treatment	60%	84%
	Prevention	29%	10%
	Combination of treatment and prevention	11%	7%
Combination of two or more antimicrobials		9%	47%

**Table 8:** Justifications for the economic advantage for antimicrobial use by farmers in a case study into antimicrobial use in broilers in Indonesia.

	No.	Percentage	Quotations
Increased productivity	108	33%	*‘Accelerate chicken growth.’* *‘Very good for chicken body weight.’* *‘Stable body weight with rising tendencies.’*
Healthier chickens	97	29%	*‘Reduce mortality.’* *‘Mortality rates can be reduced.’*
Reduced mortality	84	25%	*‘Chickens will be healthy if medicated with the right antibiotics.’*
For disease prevention	66	20%	*‘Prevent disease.’*
Treating disease	37	11%	*‘If chickens are sick, have not found any substitute for antibiotics.’*

**Table 9:** An overview of the materials and methods used to characterize the economics of antimicrobials and describe antimicrobial use behaviors and attitudes to AMR in key livestock sectors in Indonesia, Thailand, and Vietnam.

	Indonesia	Thailand	Vietnam
Production system	Small-medium commercial broiler production	Small-medium commercial pig production	Small-medium commercial pig production
Number of farms in sample	419	11	40
Location of sample farms	Central Java province (*n* = 75) West Kalimantan province (*n* = 293) Lampung province (*n* = 51)	Central region of Thailand—greater Bangkok	South—Dong Nai province (*n* = 20) North—Nam Dinh province (*n* = 20)
Number of farm visits for data collection	1	1	2
Collection of quantitative antimicrobial use data	Farmers asked to describe routine antimicrobial use Farmer recall of retrospective data over the last 2 broiler cycles	Farmers asked to describe routine antimicrobial use Farmer recall of retrospective data over the last pig cycle	Farmers asked to describe routine antimicrobial use Collection of on-farm antimicrobial use data over a six-week period through farmer’s retaining antimicrobial packaging (questionnaire conducted over two visits)
Economic antimicrobial use and productivity data	Farmer recall of retrospective data over the last 12 months	Farmer recall of retrospective data over the last 12 months	Collection of data over a six-week period (questionnaire conducted over two visits)
Qualitative data on farmer attitudes	Likert scale and open questions	Likert scale and open questions	Likert scale and open questions
Time of data collection	September 2018	October 2018	January–March 2018
Case study collaborators	FAO regional office, Directorate General of Livestock and Animal Health Services (DGLAHS) and Center for *Indonesian* Veterinary Analytical Studies (*CIVAS*)	National Institute for Veterinary Research (NIVR)	Chulalongkorn University

**Table 10:** Key findings and recommendations for policy development and implementation from the development of a framework to characterize the antimicrobial use/AMR complex in livestock systems in Indonesia, Vietnam, and Thailand.

Priority Area	Key Findings and Recommendations for Policy Development and Implementation from the Development of a Framework to Characterize the Antimicrobial Use/AMR Complex in Livestock Systems in Indonesia, Vietnam, and Thailand
Study methodology to characterize antimicrobial use	Where there are limited resources, we must focus antimicrobial use and resistance work on parts of the livestock sector with high potential for animal to human transmission of AMR. This requires knowledge of the livestock food system and capacities of the private sector to manage and implement change. Methods to obtain accurate, specific antimicrobial use data in livestock should be assessed with the analysis of the relative costs and benefits of obtaining such data. The poultry and pig sector have cycles within and between years that influence profitability and, in turn, the economic value of AMU. Therefore, meaningful antimicrobial use and resistance economic research should be carried out over a number of production cycles.
Antimicrobial use	The main antimicrobials used are penicillins, tetracyclines, and sulphonamides. Critically important antimicrobials are also frequently used. Feed labelling is sometimes misleading and does not list nutritional information or ingredients including antimicrobials added. Therefore, farmers may not know what is contained in commercially-purchased feed.
The economics of antimicrobial use	The costs of antimicrobials in the production systems are small in total and very small in relation to other costs. There is evidence that antimicrobial use in small-scale poultry production systems improves feed conversion ratios and improves overall production. The pig industry is susceptible to economic instability with falling prices placing farmers under increasing pressures to minimize costs. The poultry industry is also susceptible to economic and market stability, with day-old chick price and availability a constant concern and increasing pressure on commercial feed prices as human food demand increases. Small changes in feed prices have big impacts of cycle profitability.
Antimicrobial use and resistance policy for livestock	During the period of the study, Indonesia, Thailand, and Vietnam have embraced and implemented policies on antimicrobial use control in livestock and AMR surveillance. Thailand appears to have the most intensive, modern, and regulated livestock systems with regard to antimicrobial use and more developed policies and implementation such as HP-CIAs requiring and prescription, when compared with Indonesia and Vietnam. Thailand has quantified antimicrobial sales using the ESVAC methodology. There is potential for this methodology and approach to be adopted by other LMICs and could be applied to data already collected and supplied to OIE. Policy to regulate antimicrobial use should be stepwise with prescription only being the ultimate goal. At present, of the case study countries, Thailand appears to be further along the journey in developing antimicrobial use and resistance policy for livestock than Indonesia or Vietnam.
